# XCluSim: a visual analytics tool for interactively comparing multiple clustering results of bioinformatics data

**DOI:** 10.1186/1471-2105-16-S11-S5

**Published:** 2015-08-13

**Authors:** Sehi L'Yi, Bongkyung Ko, DongHwa Shin, Young-Joon Cho, Jaeyong Lee, Bohyoung Kim, Jinwook Seo

**Affiliations:** 1 Department of Computer Science and Engineering & Institute of Computer Technology & Bioinformatics Institute, Seoul National University, Seoul, 151-744, Korea; 2 ChunLab, Inc., Seoul National University, Seoul, 151-742, Korea; 3 Department of Statistics, Seoul National University, Seoul, 151-742, Korea; 4 Department of Radiology, Seoul National University Bundang Hospital, Gyeonggi-do, 463-707, Korea

**Keywords:** Cluster Analysis, Multiple Clustering Results, Visualization Technique, Gene Expression

## Abstract

**Background:**

Though cluster analysis has become a routine analytic task for bioinformatics research, it is still arduous for researchers to assess the quality of a clustering result. To select the best clustering method and its parameters for a dataset, researchers have to run multiple clustering algorithms and compare them. However, such a comparison task with multiple clustering results is cognitively demanding and laborious.

**Results:**

In this paper, we present *XCluSim*, a visual analytics tool that enables users to interactively compare multiple clustering results based on the *Visual Information Seeking Mantra*. We build a taxonomy for categorizing existing techniques of clustering results visualization in terms of the Gestalt principles of grouping. Using the taxonomy, we choose the most appropriate interactive visualizations for presenting individual clustering results from different types of clustering algorithms. The efficacy of *XCluSim *is shown through case studies with a bioinformatician.

**Conclusions:**

Compared to other relevant tools, *XCluSim *enables users to compare multiple clustering results in a more scalable manner. Moreover, *XCluSim *supports diverse clustering algorithms and dedicated visualizations and interactions for different types of clustering results, allowing more effective exploration of details on demand. Through case studies with a bioinformatics researcher, we received positive feedback on the functionalities of *XCluSim*, including its ability to help identify stably clustered items across multiple clustering results.

## Background

Since Eisen lab's *Cluster and TreeView *[1] popularized cluster analyses and visualizations of microarray data, cluster analysis has been widely used in the bioinformatics community. As genetic probing technologies rapidly improve in capacity and accuracy (e.g. Next Generation Sequencing), cluster analysis is playing an even more important role in the descriptive modeling (segmentation or partitioning) of the large data produced by high-throughput probing technologies. Though cluster analysis has become a routine analytic task for bioinformatics research, it is still arduous for a researcher to quantify the quality of a clustering method's clustering results.

There have been a few attempts to develop objective measures for clustering quality assessment; however, in most practical research projects, determining the quality of a clustering result is subjective and application specific [2]. To make things even more challenging, there are a large number of clustering methods, which could generate diverse clustering results. Moreover, even an individual clustering algorithm could end up with different results depending on the clustering parameters.

Since there is no generally accepted objective metric for selecting the best clustering method and its parameters for a given dataset, researchers often have to run multiple clustering algorithms and compare different results while examining the concordance/discordance among them. Such a comparison task with multiple clustering results for a large dataset is cognitively demanding and laborious.

In this paper, we present *XCluSim*, a visual analytics tool that enables users to interactively compare multiple clustering results and explore individual clustering results using dedicated visualizations.

This paper is structured as follows. In the next section we discuss some of the most relevant visualization tools and techniques, focusing on a comparative analysis of multiple clustering results. Each visualization component and its interactions in *XCluSim *are described in the Methods section. The Results and discussion section contains case studies and discussions followed by a conclusion.

### Related work

#### Visual comparison using visualizations for multi-dimensional categorical data

Since multiple clustering results can be treated as multi-dimensional categorical datasets, they can be visualized using various visualization techniques corresponding to the specific data types. These techniques include *Parallel Sets *[3] and *Parallel Coordinate Plot *[4]. Lots of prior work on the visual comparison of multiple clustering results employed these techniques [2,5-11], but we focus our discussion on the ones that are most relevant to us in terms of utilizing ribbon-like bands to represent concordance/discordance among multiple clustering results.

In *iGPSe *[5], to visually compare clustering results of two different expression data types (i.e. gene expression and micro-RNAs expression), two dimensional axes were juxtaposed, allowing for the use of parallel sets. By observing the flow of ribbon-like bands, users were easily able to see which items were shared between a pair of clusters from two different clustering results. *HCE *[2] also juxtaposed a pair of hierarchical clustering results in parallel to enable comparison tasks with the two results. In contrast to *iGPSe, HCE *used a partitioned heatmap instead of a simple node to show the details of each data item. To reveal the relations between items in a pair of heatmaps, matching items were connected with straight lines. However, these two visual analytics tools only supported the comparison of a pair of clustering results. Moreover, because they used connectivity between related items, it was often the case that there were too many crossing lines with a large dataset.

*CComViz *[6] alleviated the line crossing problem while focusing on the comparison tasks of more than two clustering results. In their work, multiple clustering results were visualized with a parallel coordinate plot: clustering results as dimensions, clusters as vertical positions in each dimension, and items as lines. Users could grasp the overall distribution of items across multiple clustering results by tracking the flow of lines crossing multiple dimensions. Similar representations were used in [7], but *CComViz *devised an algorithm for rearranging clusters and their members to minimize visual clutter between each dimension. *Matchmaker *[8] also utilized the parallel coordinate plot, but to show raw data simultaneously, partitioned heatmaps were shown in dimensional axes. The items in each dimension were rearranged by their average values so that heatmaps clearly showed the patterns of their raw data. Unlike the case of *CComViz*, in this case, partitioned heatmaps used a bundling strategy to maintain the position of each item in a dimension. This reduced line crossings between adjacent dimensions. Although this method generated a clearer overview of the distributions of items, it had some drawbacks. First of all, the flows of inner lines were invisible unless users explicitly highlighted the lines. Secondly, since the lines were bundled, the width of a band may not have accurately conveyed the number of the items belonging to the band.

*CComViz and Matchmaker *were probably most relevant to *XCluSim*. They depended on a linear ordering of dimensions (or clustering results), which made it difficult to do all-pairs comparison with a large number of clustering results at once. For example, as the authors said, *Matchmaker *only enabled users to compare, at most, six clustering results simultaneously, even with the limited linear ordering of dimensions. Since the same dataset can yield a large number of different clustering results, it is necessary to provide a more scalable way of comparing them. In *XCluSim*, we present diverse overviews to help in comparison tasks with many clustering results.

#### Visualization using similarity measures

There are a few approaches to visualize measured similarity values between clusters (or items) in different clustering results instead of explicitly visualizing shared items among multiple clustering results. Sharko et al. [12] utilized a color-coded similarity matrix view to show the stability between items or clusters across different clustering results. Similarities were measured by counting how many times each pair of items was clustered together or how many items each pair of clusters shared. Kothur et al. [13] used bar charts arranged in a matrix layout to show similarity values between a pair of clusters. However, these two works were restricted to comparing a pair of clustering results since they both used a matrix layout.

*iGPSe *[5] used *Silhouette Plot *[14] to help compare a pair of clustering results. Each item got a standardized dissimilarity value ranging from -1 to 1. This value represented dissimilarity in such a way that, when a value was close to 1, its average dissimilarity from all other items in the same cluster was much smaller than the maximum average dissimilarity from all items in another cluster. When the value was close to -1, the meaning of the value was reversed. By representing these similarity values between clustering results using a bar chart, users were able to assess the relative quality of clustering results.

These previous works using similarity measures allowed for comparisons of only a small number of clustering results. However, it is clear that, by abstracting detailed differences to simpler similarity measures, the visual comparison could be rendered more scalable. In our work, we used a graph layout and a dendrogram to show similarity overviews in a more scalable way.

#### Color encoding for clusters

Color is a powerful visual cue for representing a cluster membership. It is used in many visualization techniques, including parallel coordinate plot [6,12] and scatterplot [15-17], to discriminate clusters while revealing trends in raw data. Similar efforts exist in the visualizations of multiple clustering results. For example, when using the parallel sets view, a few distinct colors are used to encode each cluster to discriminate it from others [5,6].

However, if there are clusters from different clustering results that share the same members, it is not desirable to encode them in distinct colors since it may mislead a user into thinking that those clusters are different. Moreover, when the number of clusters increases, it is hard to color-code clusters differently, because it is hard to discriminate between more than 10 colors.

A useful color encoding strategy is *Tree Colors *[18], which was devised for tree-structured data to represent similarities between nodes. A part of the parent's hue range is recursively assigned to its child nodes. As a result, nodes with the same parent have similar colors, while those that are less similar have different colors. Moreover, this color scheme reflects the level of a node by using differentially encoded chroma and luminance in each level. If the similarities between clusters from multiple clustering results can be represented as a tree structure, *Tree Colors *may be well-suited to represent similarity among them. In *XCluSim*, we used this color scheme to color-code clusters after building a hierarchical structure by running a hierarchical agglomerative clustering (HAC) [1] with all clusters.

## Methods

### Task analysis and design goals

When performing a cluster analysis with a gene expression dataset, bioinformaticians typically follow an iterative analytics process: 1) they filter out unnecessary genes from the dataset for more focused analysis; 2) they run a clustering algorithm with the selected genes; and 3) they validate clusters in the clustering result to determine whether genes are clustered properly in the biological context. When the quality of the clustering result is not satisfactory at the validation stage, they often have to return to previous steps and run the same clustering algorithm with different parameters or run a different clustering algorithm.

Years of close collaboration with bioinfomaticians have revealed to us that they often faced challenges in this iterative analytics process. First of all, there is no flexible analytics environment that supports them through the iterative process while providing diverse clustering algorithms and keeping track of their exploration history (i.e., the sequence of the clustering algorithms and parameter settings). Moreover, it is challenging for them to effectively compare different clustering results generated during multiple iterations while investigating the quality of the results at diverse levels (i.e. clustering results level, cluster level, and gene level).

To address these challenges in the iterative process of cluster analysis, we set the following design goals for our visual analytics tool:

• To facilitate scalable visual comparison of many clustering results at diverse levels;

• To support generation of diverse clustering results;

• To promote understanding of the characteristics of each clustering algorithm and its parameters in results;

• To provide dedicated visualizations effective for different types of individual clustering results.

We designed *XCluSim *based on the *visual information seeking mantra *(i.e. overview first, zoom and filter, and details-on-demand) [19] to better support scalable visual comparison. Since each combination of different clustering algorithms and their parameters may yield different clustering results, it is inevitable from those many clustering results to 1) *see their overall similarity first*, 2) *choose a subset of them*, and then 3) *perform detail comparisons and explore individual clustering results*.

*XCluSim *provides as many clustering options as possible by implementing famous clustering algorithms and linking the clustering algorithms available in Weka [20]. It also keeps track of clustering options that users try during the analysis process.

In the following subsections, we introduce visualization techniques and user interactions for comparison tasks. They include *overview, filtering*/*selection*, and *detail view*. Then we present visualization techniques that help users to explore individual clustering results. For better comprehension of the visualization components in *XCluSim*, we first describe a color encoding strategy for clusters, which we consistently apply to every visualization component of *XCluSim *prior to explaining each visualization.

### Color encoding of clusters using *Tree Colors*

To help users identify similarities among multiple clustering results, we color-code each cluster based on *Tree Colors *[18], which provides a color-coding scheme for tree-structured data. We first hierarchically cluster all clusters from every clustering result using HAC. The correlation coefficient is used as the similarity measure between a pair of clusters as in [6]. This maintains consistency in the use of the cluster similarity measure in *XCluSim*, which is also used for rearranging bands (i.e. clusters) in the *enhanced parallel sets view *(see the Enhanced parallel sets view section). In the resulting tree-structured cluster hierarchy, we assign an appropriate color to each cluster based on the *Tree Colors *color-coding scheme so that similar clusters have similar colors.

**Figure 1:**
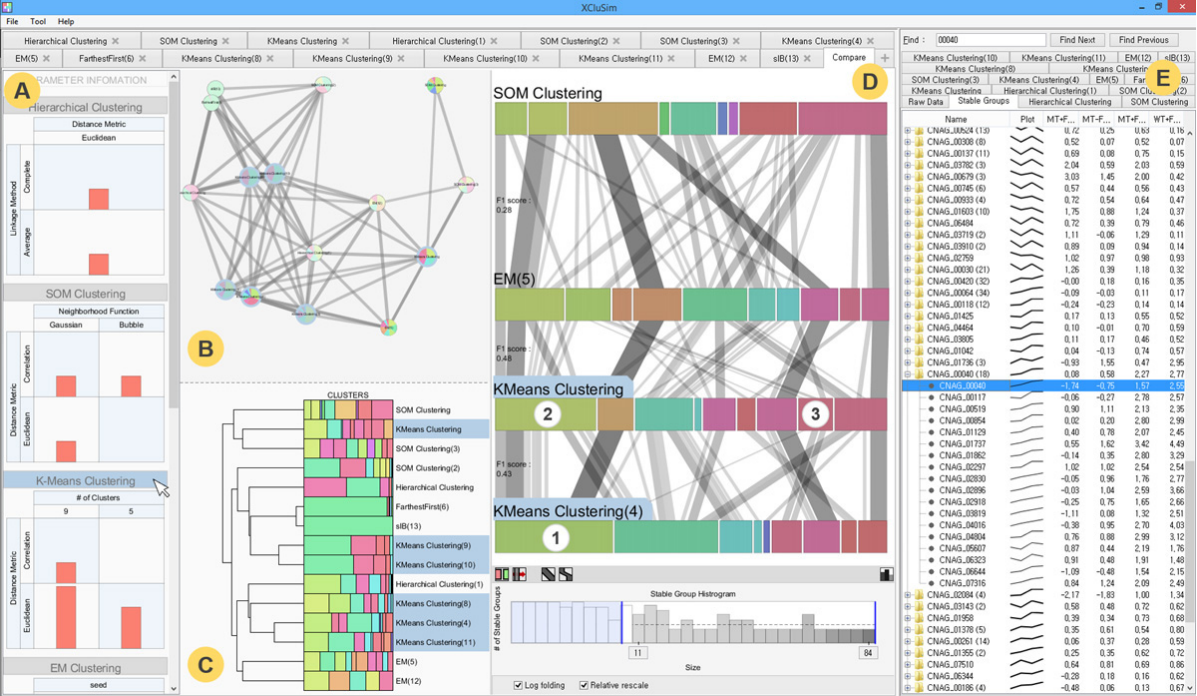
**Visualization techniques for comparing multiple clustering results in *XCluSim***. There are three types of overviews: (A) *parameter information view*, (B) *force-directed layout overview*, and (C) *dendrogram overview*. They enable users to simultaneously compare multiple clustering results in a scalable way. When some clustering results are selected in the overviews, they are added to (D) *enhanced parallel sets view* for more in-depth comparison tasks. Users can access the detailed information of the selected clustering results with each result in each tab of (E) the *tabular list view*.

This color encoding helps users intuitively assess the similarity of clusters. For example, in Figure 1D (the *enhanced parallel sets view*), **① **and **② **have very similar colors while **① **and **③ **do not, which means that **① **and **② **share most items while **① **and **③ **barely share any items. This color-coding scheme is consistently applied to overviews, detail views, and every visualization for individual clustering results.

### Overview of all clustering results

#### Parameter information view

*XCluSim *provides an overview of parameters for all clustering results in the *parameter information view *(Figure 1A, 2A). This view is vertically divided into subsections, each of which corresponds to an individual clustering algorithm (e.g. "K-means clustering"). Inside each subsection, there are multiple bar charts arranged in a matrix layout. Each bar chart shows the number of clustering results generated by the corresponding algorithm with the corresponding parameter setting. For example, in Figure 1, the *parameter information view *is divided into more than four subsections (some subsections are hidden under the scroll view) since a user made clustering results using algorithms such as HAC, self-organizing map (SOM) clustering, K-means clustering, and expectation-maximization (EM) clustering. As shown in Figure 1, the bar in the left bottom cell of K-means clustering is taller than any bars shown in any clustering algorithms, indicating that the K-means clustering algorithm with a distance measure of Euclidean distance and with 9 as the *number of clusters *is the one mostly used (Figure 1). We note here that bioinformaticians often run a clustering algorithm multiple times even with the same parameter setting when the algorithm (e.g. K-means) works non-deterministically. For more details on clustering parameters, the user can also look into the visualization of individual clustering results.

**Figure 2:**
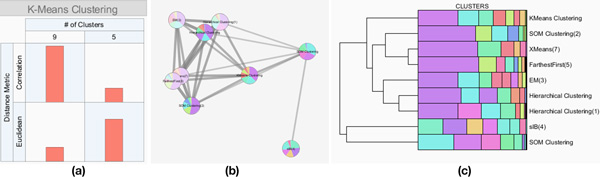
**Three overviews supported in *XCluSim***. (a) The *parameter information view* provides the parameter settings used for the clustering results produced. The table in the *parameter information view* is for a clustering algorithm, and it shows a bar in each cell to represent the number of clustering results using the corresponding parameter setting. (b) The *force-directed layout overview* intuitively shows similarity among multiple clustering results with the distance between nodes representing similarity. (c) The *dendrogram overview* shows similarities between clustering results in a familiar dendrogram layout with a clustering result visualized at a terminal node.

To help users determine which results to select for detailed analysis, *XCluSim *provides scalable similarity overviews both at the cluster level and at the clustering result level using a force-directed layout (FDL) and a dendrogram view. In the next two sections, we present details of these two overviews.

#### Force-directed layout (FDL) overview

In the *FDL overview*, overall similarity relations among multiple clustering results are visualized in a force-directed layout, where more similar results are placed closer together and connected with thicker edges (Figure 1B. 2B). The similarity metric for calculating distances between nodes is F-measure [21], which is the harmonic mean of the precision and recall measure. Each of the *precision *and *recall *measures for the two clustering results is calculated by dividing the number of agreed pairs of items by the number of all pairs of items belonging to a clustering result. An agreed pair refers to two items that "agree" to be clustered together in both clustering results.

Since the *FDL overview *uses physical distance to visually encode similarity between clusters, it has a perceptual advantage in revealing similarity relations among them. In addition, a pie chart is embedded in each node to enable users to visually estimate the number of clusters and their sizes. Since the global color encoding scheme also helps users to grasp similarities among clusters, users can estimate which clusters remain stable across different clustering results. For the scalability of the *FDL overview*, nodes become smaller as more results are added to the view. Moreover, an edge between two clusters is displayed only when similarity between the clusters exceeds a predetermined similarity threshold.

#### Dendrogram overview

The overall similarity relations are also visualized in the *dendrogram overview *(Figure 1C, 2C) after running an HAC with all clustering results (i.e. each row or node represents a result). As in the *FDL overview*, we use the F-measure as the distance measure between a pair of results. However, the visual representation and its purpose are different from the *FDL overview*. While the *FDL overview *intuitively shows similarities using physical distance, the *dendrogram overview *uses a more familiar clustering visualization component (i.e. a dendrogram) to represent similarities between clustering results. Moreover, the *dendrogram overview *is more space efficient so that users can see clustering results and cluster distributions more clearly without occlusion.

### Visualization for comparing select clustering results

When users identify clustering results of their interests in the overview of all results, they want to select them and perform more in-depth comparison with them. In the next two subsections, we introduce visualizations for comparing the selected clustering results: the *enhanced parallel sets view *and the *tabular list view*. When a user selects a result either in the *FDL *or *dendrogram overviews*, the selected result is added to the *enhanced parallel sets view *for more in-depth comparison. The *tabular list view*, located on the rightmost side of *XCluSim*, enables users to access detailed information of the selected clustering results with each result in a separate tab.

#### Enhanced parallel sets view

**Figure 3:**
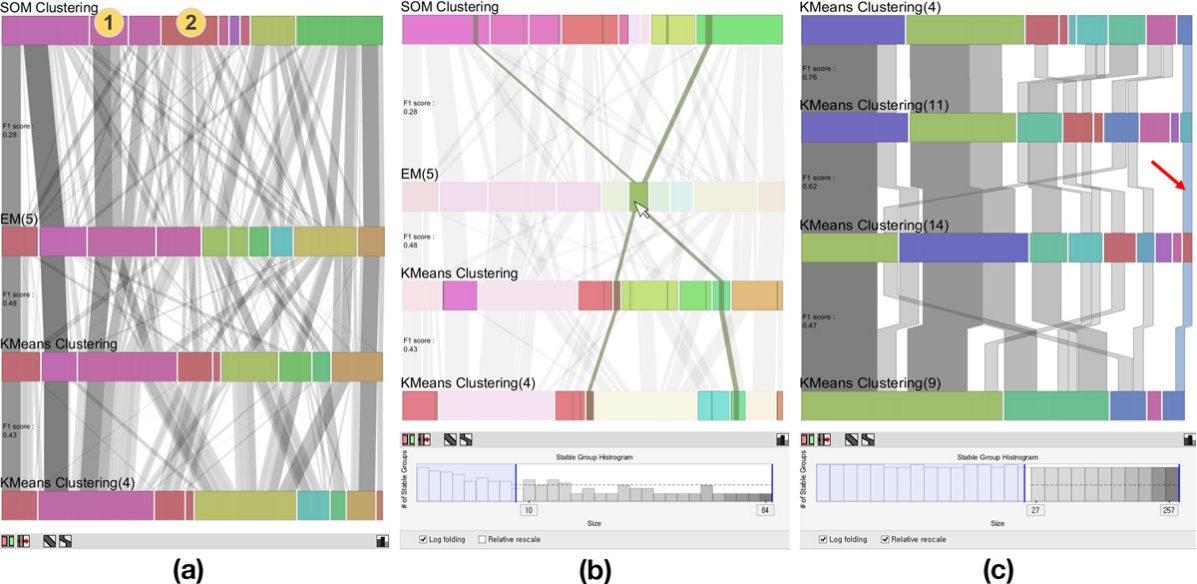
**The *enhanced parallel sets view* with various user interactions for in-depth comparison**. (a) The *parallel sets view* provides rearranging algorithms that minimize line crossings. (b) When users hover a mouse pointer over the node of a cluster, the *stable groups* contained in it are highlighted while other *stable groups* fade out to reveal flows more clearly. By using a filtering feature on the stable group histogram at the bottom of the *parallel sets view*, users can hide less interesting bands. (c) Moreover, by using *common angle plot*[22], users can compare the sizes of different bands more accurately.

To visualize the concordance and discordance of multiple clustering results in more detail, we utilized parallel sets [3]. We enhanced the parallel sets for effective clustering result comparison by designing more appropriate interactions and revealing more relevant information, i.e., *stable group *(explained in detail later in this section). In the *parallel sets view *(Figure 1D, 3), each horizontal row of stacked bars represents a clustering result. A tiny gap is placed between each bar to assist users to correctly perceive a single cluster since adjacent bars can occasionally have similar colors when the *Tree Colors *scheme is used. Rows are arranged in such a way that the distance between adjacent rows encodes the dissimilarity between the corresponding clustering results. Each horizontal bar in a row represents a cluster in the corresponding result. We define a *stable group *of items as a set of items that are clustered together through all selected clustering results. A *stable group *is represented as a ribbon-like band across all rows. Since the *parallel sets view *only enables comparisons based on a linear ordering of results, users can interactively switch any two rows by dragging one over the other. When the vertical order of the rows is changed, all rows are replaced accordingly to reflect the similarity between new adjacent clustering results.

The aggregated band representation for links connecting items in a *stable group *significantly reduces visual clutter compared to the use of a single line representation to connect individual items. The width of a band is an important visual cue that encodes important information about a *stable group *(i.e. its size) in *XCluSim*. Users can easily recognize the largest groups of items that are clustered together across multiple clustering results as they spot thick bands. Moreover, users can visually estimate the stability of a cluster by looking at the width of each *stable group *in it. For example, since the average width of *stable groups *in **① **is bigger than **② **in Figure 3A, a user can infer that **① **is a more stable cluster than **②**. Cluster-similarity based on the color-coding of bars (i.e. clusters) helps to facilitate the comparison of multiple clustering results.

However, the aggregation method could still suffer from clutter due to band-crossings. We applied a rearrangement algorithm [6] to address this issue. To provide more flexible user interaction depending on a user's need, we divided the algorithm into two rearrangement features: rearranging clusters (i.e. bar rearrangement) and rearranging their members (i.e., band rearrangement). These features can be evoked by pressing on the button at the bottom of the enhanced parallel sets view (Figure 1D). When a user uses any of these two features, smooth animated transition is supported to reduce the cognitive burden that accompanies users' attempts to trace the movement of bands or bars.

*XCluSim *provides more user interactions to overcome the cluttering problem. First of all, users can alleviate the visual clutter in the region of interest by rearranging the bars in a row. This involves dragging them horizontally. After manually rearranging bars (i.e. clusters), users can employ the band rearrangement feature to reduce the visual clutter of bands across multiple rows due to the current manual arrangement of bars in the row. Secondly, there is a band filtering feature similar to that in [11]. The *stable group *histogram at the bottom of Figure 3C shows the distribution of bands by size. There are two blue filtering bars on both sides. Users can filter out bands that are too small or too big from the *parallel sets view *by adjusting the position of the filtering bars. Finally, when the mouse pointer hovers over a cluster, it highlights the bands, allowing the clusters to show their flows across other clustering results clearly (Figure 3B). This can be helpful when a user is especially interested in *stable groups *that belong to a specific cluster.

The perception of a *stable group's *size could be distorted by a *line width illusion *[22]. Such an illusion causes humans to perceive line width incorrectly at slanted angles. This distortion may disrupt the task of band size comparison. In order to prevent it, we adopt the *common angle plot *[22] idea (Figure 3C). By comparing the straight, vertical parts of bands, users can compare the sizes of the *stable groups *more accurately. However, since the *common angle plot *represents a single line as three connected straight lines, it may generate more clutter and occlusions. Thus, it is better to use this feature when only a small number of bands are displayed in the *parallel sets view*.

#### Tabular list view

**Figure 4:**
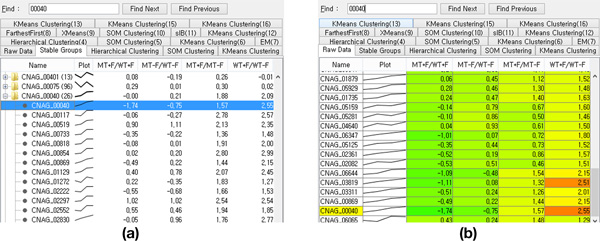
**The *tabular list view* enables users to access numerical details**. (a) Users can see detailed information for each item grouped by *cluster* or *stable group*. (b) Users also can see raw data in a heatmap form. When a user wants to access an item or a group directly, he/she can use the search box provided on top of the *tabular list view*.

Users can access detailed information concerning the selected clustering results with each result in a separate tab in the *tabular list view *(Figure 4). The tabular view provides detailed information in two different modes: the group-by mode and the heatmap mode. In the group-by mode, users can see the data grouped by *stable groups *or by clusters. A group is represented by a representative item in a single row with the number of group members between parentheses. Moreover, there is a line graph glyph in each row to show the overall average pattern of the corresponding group. In the heatmap mode, the *tabular list view *shows numerical details with each cell color-coded according to its value. There is a text search field on top of the *tabular list view *so that users can directly access specific items. A user can export a selected subset of data (e.g. a specific *stable group*) as a CSV text file for further analysis.

*XCluSim *provides brushing and linking among all visualization components. Thus, the *tabular list view *is coordinated with all visualization components in *XCluSim*. Thus, whenever a user selects a group of items in any visualization, they are highlighted in the *tabular list view *to help the user access detailed information about them. In addition, when the mouse pointer hovers over an item in a component, it highlights the item in white-blue color, and all related items on the other components are also highlighted. This could lead to additional meaningful insights. For example, hovering a mouse pointer over the title of a specific algorithm in the *parameter information view *results in the highlighting of all related clustering results in overviews and detail views (Figure 1). As a consequence, users are able to understand that K-means clustering can produce totally different clustering results depending on the clustering parameters chosen (e.g. compare "K-means clustering(10)" to "K-means clustering(11)" in the *dendrogram overview *in Figure 1).

### Interactive data manipulation

**Figure 5:**
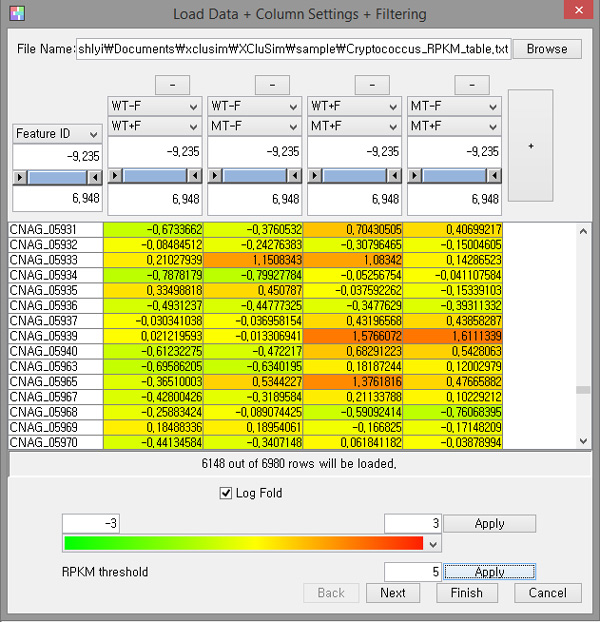
**Interactive manipulation of input data supported by *XCluSim***: derive a new column (ratio, fold change), change color mapping, filter items using a range filter and RPKM adjustment.

Simple file formats such as comma separated values (CSV) and tab-delimited text are used for *XCluSim. XCluSim *enables researchers to interactively manipulate the input dataset when loading it, prior to clustering it (Figure 5). Users can generate a ratio value by selecting two columns from the original dataset. *XCluSim *provides filters such as a range filter and RPKM threshold adjustment. It also provides features for calculating fold changes.

### Visualization for individual clustering results

To make *XCluSim *a more general visual analytics tool for comparing clustering results, we try to provide a wide variety of clustering algorithms. First of all, we implement frequently-used clustering algorithms in *XCluSim*. These include Hierarchical Agglomerative Clustering [1], SOM clustering [23], K-means clustering, and OPTICS clustering [24]. Moreover, all clustering algorithms from Weka [20] are also available in *XCluSim*. Users can also import any clustering results made by any other clustering algorithms that are not available in *XCluSim*.

#### Taxonomy of visualization techniques for visualizing clustering results

Different clustering algorithms work on different principles. For example, there are three major categories of clustering algorithms: hierarchical, partitional, and density-based. Clustering algorithms in different categories need different visualization techniques to effectively visualize their clustering results.

To suggest effective visualizations for each category of clustering algorithms, we first surveyed visual encoding techniques for visualizing the clustering results of various algorithms (Table 1). Sedlmair et al. presented a related taxonomy of factors in visual cluster separation [28]. They evaluated the effect of each factor on visual cluster separation in scatterplots. Building upon this work, we consider the appropriateness of visual encoding techniques in representing the characteristics of each type of clustering algorithm. To broaden the perspective of our taxonomy, we further categorize the visual encoding techniques in terms of Gestalt principles of grouping [27]: similarity, proximity, connectedness, and enclosure.

**Table 1:** Taxonomy of visualization techniques for visualizing clustering results.

Principle to Show Cluster Membership		Visualization Component	Clustering Algorithm		
		
Main	Secondary		Hierarchical	Partitional	Density-based
		
Similarity (color or size)	Proximity	Scatterplot + Color	Δ	Δ [17]	Δ [16]
		
		*Graph (vertex as item) **+ Color**	Δ	Δ [5]	Δ [16]
		
		*Bar chart (Reachability Plot)	X	X	O [15,20,24]
	
	Enclosure	Colored shape	Δ [25]	Δ	Δ [13]
	
	.	*Parallel coordinate plot + Color	Δ [6]	Δ	Δ
**Proximity**	Similarity and Enclosure	**Bar chart **(Silhouettes Plot)	Δ	O [5,14]	O

**Connectedness** (line connection)	Similarity and Proximity	*Dendrogram	O [2,8,9]	X	Δ
		
		**Normal tree**	Δ [26]	X	Δ
		
		**Circular tree**	Δ [26]	X	Δ

**Enclosure**	Proximity	*Heatmap + Partitioning	O	O	O
	
	Similarity and Proximity	**Treemap**	Δ [26]	X	X

*Visualization techniques supported in *XCluSim*					

Visualization components for visualizing clustering results use visual cues based on Gestalt principles of grouping [27] to represent cluster membership. We categorize the visualization components by principle and indicate how appropriate each visualization component is for showing clustering results by different types of clustering algorithms. (i.e. "O" for most appropriate, "Δ" for moderately appropriate, and "X" for not applicable).

**Similarity: **The similarity principle is the one most commonly used in cluster visualization. It helps users to perceive cluster membership by employing similar colors, shapes, or sizes. Among them, color is the most frequently used visual cue. However, using color as the main visual cue may not scale well because the use of human color perception to discriminate between classes is limited to a number of colors. Thus, it is often used in conjunction with visual cues such as in reachability plot [20] and silhouettes plot [5].

**Proximity: **This principle facilitates the perception of cluster membership by placing related items closer together. For example, in the silhouettes plot [14], bars belonging to the same cluster are placed next to each other. However, this principle is not used alone. It is typically used together with other visual cues. For example, the partitioned heatmap sometimes puts gaps between clusters to show their boundaries clearly [2,8,10,11].

**Connectedness: **The connectedness principle helps users to identify groups by connecting related items using a visual artifact such as a line. Line connection is one of the most powerful visual cues among the Gestalt principles of grouping. However, it can confuse users when there are too many lines in a single view. The connectedness principle is especially used with hierarchical clustering results since hierarchy structures can best be demonstrated with line connections. For example, *HCE *[2], *Matchmaker *[8], and others use this principle to represent clusters in dendrograms.

**Enclosure: **The enclosure principle is adopted particularly when drawing a closed boundary containing items belonging to a cluster. For example, when a dataset contains spatial information, all items of a cluster are shown on a color-coded region with a solid boundary [13,25]. Another typical technique based on this principle is the partitioned heatmap [10,11]. It is a powerful way to display raw data while clearly specifying the boundary surrounding the members of each cluster.

In addition to these four Gestalt principles of grouping, there are some attempts to use abstract representations (such as glyphs or special shapes) for clusters without showing any individual items in clusters. The cluster graph [29] uses an abstract representation of a circular node for a cluster. Clusters derived from SOM clustering results are visualized in a hive-shaped grid view while each item is abstracted as a node [23]. As these attempts do not allow for the visualization of individual items, they are not a good fit for the classification based on Gestalt principles.

After reviewing and categorizing visual encoding techniques for visualizing clustering results, we selected visualization techniques appropriate for visualizing each of three main kinds of clustering algorithms (namely, hierarchical clustering, partitional clustering, and density based clustering). In the next three subsections, we describe the visualization techniques in detail.

#### Visualization technique for hierarchical clustering

**Figure 6:**
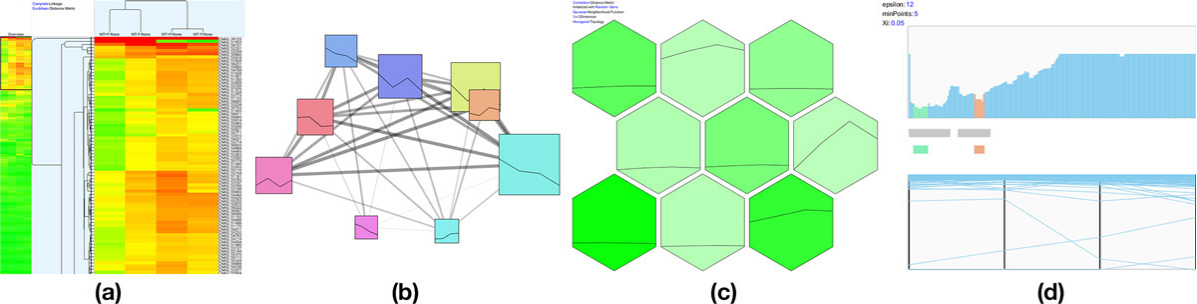
**Visualization techniques for individual clustering results in *XCluSim***. (a) *Dendrogram+heatmap* visualization for hierarchical agglomerative clustering results. (b) Force directed layout for every partitional clustering result and imported clustering results. (c) Common hive-shaped visualization for SOM clustering results. (d) Reachability plot together with parallel coordinate plot for OPTICS.

We visualized HAC results with the combination of a dendrogram and heatmap visualization (Figure 6A), where users could interactively compress/expand, flip, and swap sub-trees. The batch compression of sub-trees using the minimum similarity bar [2] is also possible. By adjusting the position of the similarity bar, users can dynamically determine the clusters. There is a compact bird's-eye overview using heatmap [30] in the left most part which is tightly coupled with the dendrogram. By dragging a black-bordered rectangle that represents the current viewport (see the black rectangle in the top left of Figure 6A) in the heatmap overview, users can efficiently navigate through the *dendrogram+heatmap *view.

#### Visualization technique for partitional method

Partitional clustering results other than SOM clustering (e.g. K-means clustering, EM clustering, farthest first clustering, etc.), and all imported results are visualized in a force-directed layout (Figure 6B), where each cluster is represented as a rectangle whose size is proportional to the cluster size. The force between nodes is determined by the similarity between members of each cluster so that similar clusters are closely positioned and have thicker links between them. To show an overview of a cluster, *XCluSim *also visualizes the average pattern of all members of the cluster in a line chart, which is shown as a glyph in the cluster's node. *XCluSim *also supports semantic zooming to enable users to explore clusters in more detail. When a cluster is zoomed into, more details of its members are dynamically visualized in a parallel coordinate plot.

SOM clustering results are visualized using the typical hive-shaped visualization (Figure 6C), where each hexagonal cell represents a cluster. In *XCluSim*, the background intensity of each cell represents the size of the corresponding cluster. As a visual summary of each cluster, *XCluSim *presents the average pattern of the cluster members in a line chart within each hexagonal cell. *XCluSim *also supports semantic zooming. Users can zoom into a cluster by double-clicking on the corresponding cell and look at the details of their members in a parallel coordinate plot in the same way they would in a force-directed layout.

#### Visualization technique for density-based method

Density-based clustering algorithms calculate a kind of density-related information for each item during the clustering process. For example, OPTICS [24] calculates the reachability distance for each item. We believe that users can more intuitively understand a density-based clustering result when the density-related information is revealed. Therefore, a bar-chart-like visualization, with each item arranged on the horizontal axis and the density-related information on the vertical axis, can effectively visualize density-based clustering results. The conventional reachability plot for OPTICS is a typical example. In *XCluSim*, we enhance the plot for better cluster identification and for improved examination of details (Figure 6D). To clearly show the position of each cluster, *XCluSim *places a horizontal bar from the start to the end positions of the cluster right below the reachability plot. The parallel coordinate plot at the bottom shows more details of cluster members. These two plots support brushing and linking between the cluster members. For example, when a mouse pointer hovers over a cluster in the reachability plot, the lines for the members of the cluster are highlighted in the parallel coordinate plot.

### Implementation

*XCluSim *was developed using Java Standard Edition 7 (Java SE 7), which enables it to run on any platform with JRE version 1.7 or higher. We used the Piccolo 2D framework to implement visualization components and interactions. Weka's clustering algorithms were integrated into *XCluSim *using Weka SDK 3.6 [20].

## Results and discussion

### Case studies

To evaluate the efficacy of *XCluSim*, we conducted two case studies with our collaborator in a major bioinformatics research laboratory. He is a senior research engineer and has years of experience in genome and transcriptome analyses.

#### Elucidating the role of ferroxidase in cryptococcus neoformans var. grubii H99 (case study 1)

This study was carried out in his laboratory for 80 minutes. Pre- and post-study interviews were conducted for 10 minutes each. The participant used *XCluSim *for 50 minutes after a 10-minute tutorial. We used a dataset containing normalized expression levels of 6,980 genes belonging to the *Cryptococcus neoformans var. grubii H99 *strain. The dataset had been prepared for his previous work [31].

His task was to elucidate the role of ferroxidase (cfo1) by knocking it out. He was interested in finding a meaningful set of genes whose expression would be influenced and in identifying the affected pathways. For the task, he tried to see the effect of fluconazole on two different strains: the wild type of *Cryptococcus neoformans var. grubii H99 *and the cfo1 mutant of the same strain. In the dataset, each gene has four expression levels: two different strains, each cultured in two conditions (i.e. wild-type strain and cfo1 mutant with and without fluconazole treatment).

When he loaded the data, he made four new data columns of ratio values, including the wild-type strain with fluconazole versus the wild-type strain without fluconazole treatment (WT+F/WT-F) and the cfo1 mutant with fluconazole versus the cfo1 mutant without fluconazole treatment (MT+F/MT-F) (Figure 5). Subsequently, he adjusted the RPKM threshold and used log fold changes to filter out less interesting genes for more efficient analysis.

After data pre-processing, *XCluSim *showed the results of three clustering algorithms (i.e. HAC, SOM clustering, K-means clustering) in three independent views. Since he was most familiar with dendrogram and heatmap visualization, he examined the HAC results first. He was interested in genes that were highly expressed with fluconazole treatment. Among them he found the gene named Erg11 (CNAG_00040). He said that this gene was reported to be associated with azole resistance.

Next, he tried to see which genes were stably grouped together across different clustering results. He tried to load as many clustering results as possible to see the differences between them. The *parameter information view *provided him with a good overview of all clustering results (clustering algorithms and their parameters). He was able to make diverse clustering results without generating any duplicate results.

After generating 15 different clustering results, he selected four diverse results from the *FDL overview *to find out which genes were clustered together with Erg11. However, he recognized that the *stable groups *were excessively thin because of the result named "FarthestFirst(6)." This had to do with the fact that it was the most dissimilar result to other selected clustering results (Figure 1). So he removed that result from the *parallel sets view*. Then he selected a more similar one named "KMeans Clustering(4)" (Figure 3A). He subsequently accessed the *stable group *with Erg11 directly, utilizing the search feature in the *tabular list view*. He was able to confirm that 17 other genes belonged to the *stable group*. After validating the members of the *stable group *with an enrichment analysis, he found that most of them (10 out of 18) belonged to the ergosterol biosynthetic pathway.

Once he had selected the *stable group *in the *tabular list view*, he was able to efficiently inspect the flow of the group across different clustering results in the *enhanced parallel sets view *(Figure 3B). While he looked into the flow of the *stable group *across all rows (the rightmost highlighted-band in Figure 3B), he also noticed that the clustering result from "KMeans Clustering(4)" had the tightest cluster, which included the *stable group*. However, there were no more genes outside the *stable group *in the cluster that belonged to the ergosterol pathway.

Then he tried to find the best algorithm and those of its parameters that gave the tightest cluster containing genes belonging to the ergosterol pathway. Since "KMeans Clustering(4)" had previously been the best clustering result among the selected results, he ran K-means clustering algorithms with different parameters to arrive at similar results. He then inserted three of the most similar results in the *parallel sets view *(Figure 3C). Again, he highlighted a *stable group *with Erg1 (the band indicated with a red arrow in Figure 3C). By checking the flow of the stable group crossing each result, he recognized that "KMeans Clustering(14)" gave the tightest cluster. This led to the conclusion that K-means clustering with the corresponding parameter configurations (i.e. Euclidean distance as the *distance metric *and 9 as the *number of clusters*) was the best result for the given dataset among all the results.

#### Finding a clustering result that clearly represents biological relations (case study 2)

A second case study was subsequently carried out with the same participant in his laboratory. The study was conducted for 150 minutes on a different day. Since the participant was already familiar with *XCluSim*, we skipped the tutorial. In the study, he relied on the gene expression profiles of 169 genes in *Escherichia coli*, which used a DNA microarray [32]. In the dataset, each gene contained 19 expression levels in order to investigate the effects of the perturbations on tryptophan metabolism. The expressions were measured under the following conditions: wild type growth with and without tryptophan (five conditions), wild type growth with and without tryptophan starvation (nine conditions), and the growth of wild type and a *trp *repressor mutant (five conditions).

Through the case study, the participant wanted to find a clustering result that clearly reflected biological relations in tryptophan metabolism. In the original paper [32], the authors used HAC to cluster the 169 gene expression profiles measured in the 19 conditions. It was indicated in the paper that genes showing similar expression responses did not necessarily fall into the same cluster. One example included the genes associated with aromatic amino acid metabolism.

**Figure 7:**
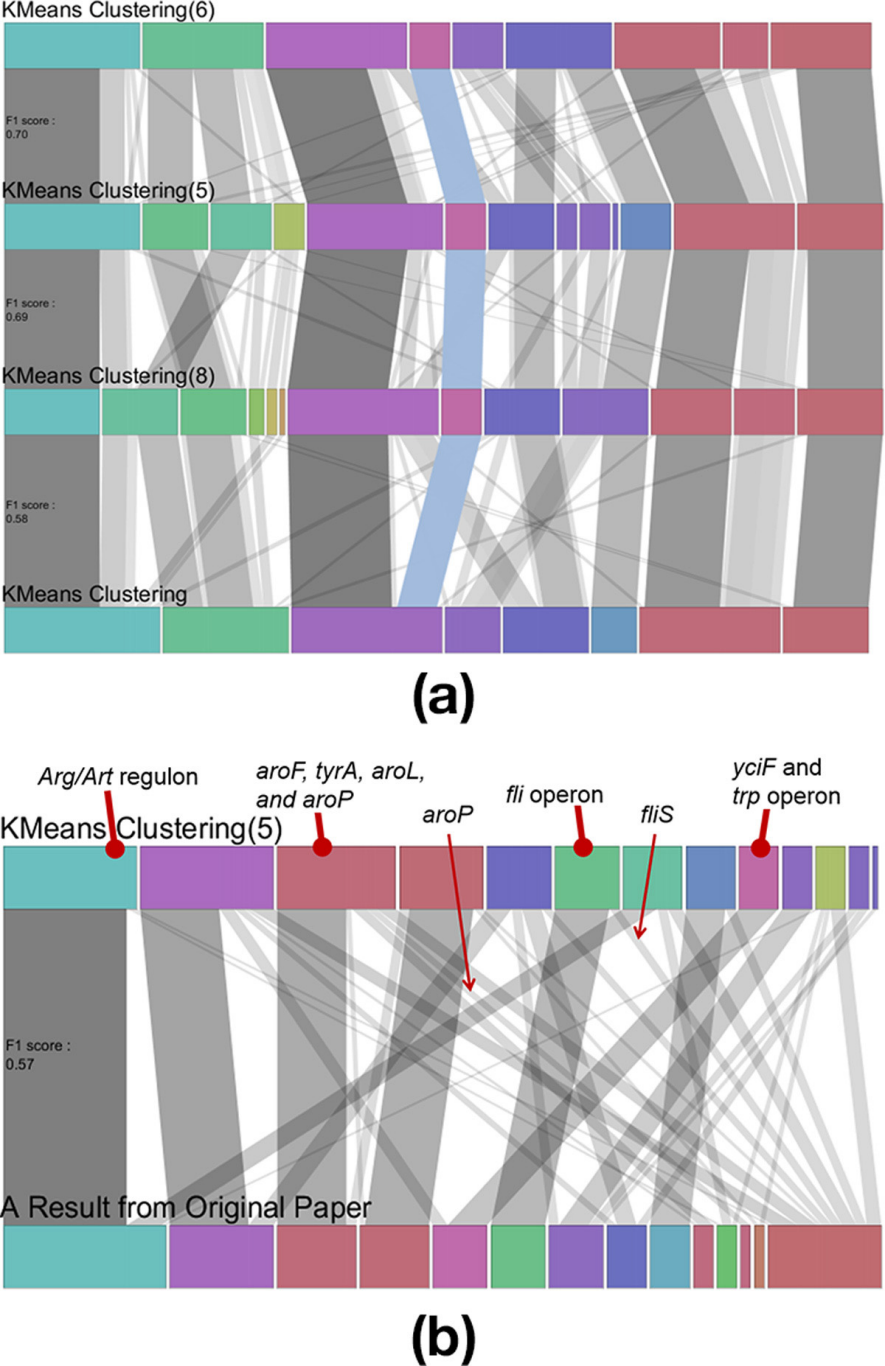
**Results of the second case study are visualized in the *enhanced parallel sets view***. (a) The highlighted *stable group* contained the *trp* operon with *yciF*. (b) Visual comparison of two results: the best clustering result ("KMeans Clustering(5)") derived from the case study and a result ("A Result from Original Paper") presented in the original research paper [32].

He first wanted to see if the optimal algorithm and its parameters in the previous case study would work for another dataset. To determine this, he produced 11 clustering results in *XCluSim*, including the result produced using previous optimal settings: K-means clustering with Euclidean distance as the *distance metric *and 9 as the *number of clusters*. He validated each cluster in the result ("KMeans Clustering(6)" in Figure 7A) through an enrichment analysis using the DAVID website (http://david.abcc.ncifcrf.gov/). After validating each cluster, he concluded that most of the clusters were grouped well in the sense that they represented biological relations in pathways. However, he recognized two problems in the result. First of all, a cluster that had both *Arg *and *Art *regulons also contained a gene named *tnaA *that was considered to be noise. This was because *tnaA *showed a different expression pattern and was not highly related to other cluster members in biological terms. Secondly, one gene from the *fli *operon, *fliS*, fell into a different cluster from the other genes in the same operon while they had homogeneous expression patterns.

By utilizing visualizations in *XCluSim*, he wanted to find the clustering result that properly represented biological relations as "KMeans Clustering(6)" while the two problems were revisited. For this intended task, he selected all the similar results from the *FDL overview*: "KMeans Clustering(5)", "KMeans Clustering(8)", and "KMeans Clustering". Then he accessed the *stable groups *that contained *tnaA *and the *Arg/Art *regulon. He easily recognized that genes in both the *Arg *and *Art *regulons fell into same *stable group *while *tnaA *was not stably clustered with them. The results, which separately clustered *tnaA *from the *Arg *and *Art *regulons, were "KMeans Clustering(5)" and "KMeans Clustering(8)". Similarly, by checking the flow of *stable groups *in each horizontal row, he easily recognized that two clustering results that used the correlation coefficient as a *distance metric *clustered two *stable groups *together: one with the *fli *operon and the other with *fliS*. The two results were "KMeans Clustering(5)" and "KMeans Clustering". As a consequence, "KMeans Clustering(5)", using the correlation coefficient as the *distance metric *and 13 as the *number of clusters*, was the most satisfying result for the dataset.

Additionally, our participant gained insight by seeing a *stable group *in *XCluSim*. Genes in the *trp *operon (i.e. *trpE, trpD, trpC, trpB*, and *trpA*) were stably clustered together with *yciF *through the four different results (see the highlighted *stable group *in Figure 7A). Since *yciF *was assigned to a putative function, he said that the gene might be closely related to tryptophan synthase as a *trp *operon.

After he found the best result, he compared it with a clustering result provided in the original work [32] to see if his result better represented biological relations (Figure 7B). The clustering result presented in the paper had been prepared prior to the study and was imported to *XCluSim *for visual comparisons. After comparing two results, he found that some of the genes involved in aromatic amino acid metabolism, *aroF, tyrA, aroL*, and *aroP*, were clustered together in our best result while only three of them fell into the same cluster in their original result. Moreover, their result did not cluster *fliS *with the other *fli *operon. These results suggested that the authors of the original work [32] could have generated more biologically meaningful results if they had used *XCluSim *in the first place.

## Discussion

During the case studies, we received positive subjective feedback on *XCluSim *from the participant. He especially liked the ability to identify *stable groups *across multiple clustering results. Moreover, he was satisfied that he could select and run diverse clustering algorithms and interactively compare them by adding/removing a clustering result to/from the *enhanced parallel sets view*. He could quickly shift his attention to a more interesting set of results for more in-depth comparison. However, he also pointed out the limitations of *XCluSim*. Since filtering sets of items was only available at the data manipulation step, he said it would be helpful to allow users to interactively filter raw data in the visualization components as well.

We color-coded each cluster consistently across the whole system using the *Tree Colors *scheme after building a hierarchical structure of all clusters from multiple clustering results. With the help of this color coding, overviews became even more useful in *XCluSim*. While the color encoding was applied for a specific purpose in this work (i.e. for the visualization of clusters), we think it can also be applied to parallel sets applications in a more general and scalable way. For example, instead of distinguishing only a small number of categories while visualizing a categorical dataset, it might be possible to distinguish many more nodes in the parallel sets once a hierarchical structure of the nodes has been built in a similar manner to the one we employed in *XCluSim*.

We provided a taxonomy of visualization techniques for visualizing clustering results based on the Gestalt principle of grouping and the types of clustering algorithms. The design space defined by this taxonomy can help researchers to make design decisions for clustering results visualization. By thinking about visualization techniques in terms of the Gestalt principle, researchers can come up with better visual encoding without overlooking important features. For example, since the graph layout is used to visualize cluster memberships by color-coding each item [5,16], one can also utilize the enclosure principle (like *GMap *[33] and *BubbleSets *[34]) to represent their membership more clearly.

## Future work

At present, when a clustering algorithm does not assign all items to clusters, all un-clustered items are treated as a single cluster in *XCluSim*. OPTICS and DBSCAN clustering algorithms can give rise to results of this kind. *XCluSim *treats un-clustered items as a group of less interesting items as if it were a special cluster. Otherwise, it could make a huge number of *stable groups *since each un-clustered item will become a single *stable group*. This would make it hard for users to gain insight from visualizations. In the future, we plan to improve *XCluSim *to resolve this problem. For example, we can represent these kinds of groups with different textures in the *parallel sets view *to distinguish them from other normal clusters.

In this paper, we concentrated mostly on supporting comparison tasks based on the concordance/discordance of multiple clustering results. However, since bioinformaticians' cluster analysis is highly integrated with the validation stage, it would also be valuable to provide a visual representation of cluster validity measures (e.g. internal cluster validity indices). For example, the gray scale intensity of each band (i.e. *stable group*) in the *parallel sets view*, which currently represents the size of a *stable group*, can be utilized to represent its internal validity measures. In such a case, *stable group *provided by *XCluSim *will become more reliable information.

## Conclusion

In this paper, we presented *XCluSim*, a visual analytics tool that enables users to compare multiple clustering results. *XCluSim *provides three different overviews to help users grasp their overall similarity relationships in a more scalable and flexible way. Moreover, the *enhanced parallel sets view *enables users to detect differences among select clustering results even more clearly by using improved user interactions. To help users not only compare but also explore individual clustering results more effectively, we proposed dedicated visualizations for each of the three distinctive classes of clustering algorithms. To design them, we defined a design space for clustering results visualization by building a visualization taxonomy based on the Gestalt principles of grouping. This taxonomy could be useful for other researchers when they design new visualizations for a clustering result. We conducted case studies to evaluate the usefulness of *XCluSim*, and the participants gave positive feedback.

## List of abbreviations

HAC: Hierarchical agglomerative clustering; SOM: Self-organizing map; EM: Expectation-maximization; FDL: Force-directed layout.

## Competing interests

The authors declare that they have no competing interests.

## Authors' contributions

JS and BK (Kim) designed the study. SL and BK (Ko) developed *XCluSim*. DWS implemented OPTICS algorithm and its visualization in *XCluSim*. YJC participated in the system design and case studies. JS, BK (Kim), and JL provided guidance and critical insights into visualization techniques in *XCluSim*. SL and BK (Ko) drafted the manuscript. All authors contributed to writing and editing the manuscript. All authors read and approved the final manuscript.

## Declarations

The publication fees and Open Access expenses for this article were funded by an academic activity support program of Seoul National University.

This article has been published as part of *BMC Bioinformatics *Volume 16 Supplement 11, 2015: Proceedings of the 5th Symposium on Biological Data Visualization: Part 1. The full contents of the supplement are available online at http://www.biomedcentral.com/bmcbioinformatics/supplements/16/S11

## References

[B1] Eisen MB Spellman PT Brown PO Botstein D Cluster analysis and display of genome-wide expression patterns Proc Nat Acad Sci 1998 95 25 14863 14868 10.1073/pnas.95.25.14863 9843981 PMC24541

[B2] Seo J Shneiderman B Interactively exploring hierarchical clustering results Computer 2002 35 7 80 86

[B3] Kosara R Bendix F Hauser H Parallel sets: visual analysis of categorical data IEEE Trans Vis Comput Graph 2005 12 4 558 568 16805264 10.1109/TVCG.2006.76

[B4] Inselberg A Dimsdale B Parallel coordinates: a tool for visualizing multi-dimensional geometry Visualization, 1990. Visualization '90., Proceedings of the First IEEE Conference on 1990 361 378

[B5] Ding H Wang C Huang K Machiraju R iGPSe: A visual analytic system for integrative genomic based cancer patient stratification BMC Bioinformatics 2014 15 203 10.1186/1471-2105-15-203 25000928 PMC4227100

[B6] Zhou J Konecni S Grinstein GG Visually comparing multiple partitions of data with applications to clustering SPIE Proceedings 2009 7243 72430J 10.1117/12.810093

[B7] Havre SL Shah A Posse C Webb-Robertson BJ Diverse information integration and visualization Proc SPIE 2006 6060 60600M 10.1117/12.643492

[B8] Lex A Streit M Partl C Kashofer K Schmalstieg D Comparative analysis of multidimensional, quantitative data IEEE Transactions on Visualization and Computer Graphics 2010 16 6 1027 1035 20975140 10.1109/TVCG.2010.138

[B9] Pilhofer A Gribov A Unwin A Comparing clusterings using Bertin's idea IEEE Transactions on Visualization and Computer Graphics 2012 18 12 2506 2515 10.1109/TVCG.2012.207 26357159

[B10] Lex A Streit M Schulz HJ Partl C Schmalstieg D Park P Gehlenborg N StratomeX: visual analysis of large-scale heterogeneous Genomics data for cancer subtype characterization Comput Graph Forum 2012 31 3pt3 1175 1184 10.1111/j.1467-8659.2012.03110.x PMC5145272 27942089

[B11] Lex A Schulz H Streit M Partl C Schmalstieg D VisBricks: multiform visualization of large, inhomogeneous data IEEE Transactions on Visualization and Computer Graphics 2011 17 12 2291 2300 22034349 10.1109/TVCG.2011.250

[B12] Sharko J Grinstein GG Marx KA Zhou J Cheng CH Odelberg S Simon HG Heat map visualizations allow comparison of multiple clustering results and evaluation of dataset quality: Application to microarray data Information Visualization 2007 521 526

[B13] Kothur P Sips M Dobslaw H Dransch D Visual Analytics for Comparison of Ocean Model Output with Reference Data: Detecting and Analyzing Geophysical Processes Using Clustering Ensembles IEEE Trans on Vis and Comput Graph 2014 20 12 1893 1902 10.1109/TVCG.2014.2346751 26356903

[B14] Rousseeuw PJ Silhouettes: a graphical aid to the interpretation and validation of cluster analysis Journal of Computational and Applied Mathematics 1987 20 53 65

[B15] Kandogan E Just-in-time annotation of clusters, outliers, and trends in point-based data visualizations Visual Analytics Science and Technology (VAST), 2012 IEEE Conference on 2012 73 82

[B16] Andrienko G Andrienko N Rinzivillo S Nanni M Pedreschi D Giannotti F Interactive visual clustering of large collections of trajectories Visual Analytics Science and Technology, 2009. VAST 2009. IEEE Symposium on 2009 3 10

[B17] Hossain MS Ojili PKR Grimm C Muller R Watson LT Ramakrishnan N Scatter/gather clustering: Flexibly incorporating user feedback to steer clustering results IEEE Trans on Vis and Comput Graph 2012 18 12 2829 2838 10.1109/TVCG.2012.258 26357192

[B18] Tennekes M de Jonge E Tree Colors: Color Schemes for Tree-Structured Data IEEE Trans on Vis and Comput Graph 2014 20 12 2072 2081 10.1109/TVCG.2014.2346277 26356921

[B19] Shneiderman B The eyes have it: A task by data type taxonomy for information visualizations Visual Languages, 1996. Proceedings., IEEE Symposium on 1996 336 343

[B20] Hall M Frank E Holmes G Pfahringer B Reutemann P Witten IH The WEKA data mining software: an update SIGKDD Explor Newsl 2009 11 1 10 18 10.1145/1656274.1656278

[B21] Van Rijsbergen CJ Foundation of evaluation Journal of Documentation 1974 30 4 365 373 10.1108/eb026584

[B22] Hofmann H Vendettuoli M Common angle plots as perception-true visualizations of categorical associations IEEE Trans on Vis and Comput Graph 2013 19 12 2297 2305 10.1109/TVCG.2013.140 24051796

[B23] Kohonen T Self-Organizing Maps Berlin/Heidelberg, Germany: Springer 1995 30

[B24] Ankerst M Breunig M M Kriegel HP Sander J OPTICS: ordering points to identify the clustering structure ACM Sigmod Record 1999 28 2 49 60 10.1145/304181.304187

[B25] Packer E Bak P Nikkila M Polishchuk V Ship HJ Visual analytics for spatial clustering: Using a heuristic approach for guided exploration IEEE Trans on Vis and Comput Graph 2013 19 12 2179 2188 10.1109/TVCG.2013.224 24051784

[B26] Beham M Herzner W Groller ME Kehrer J Cupid: Cluster-based Exploration of Geometry Generators with Parallel Coordinates and Radial Trees IEEE Trans on Vis and Comput Graph 2014 20 12 1693 1702 10.1109/TVCG.2014.2346626 26356883

[B27] Ware C Information Visualization: Perception for Design Morgan Kaufmann; 1999 180 199

[B28] Sedlmair M Tatu A Munzner T Tory M A taxonomy of visual cluster separation factors Computer Graphics Forum 2012 31 3pt4 1335 1344 10.1111/j.1467-8659.2012.03125.x

[B29] Wang W Wang H Dai G Wang H Visualization of large hierarchical data by circle packing Proc of the SIGCHI 2006 517 520

[B30] Lex A Streit M Kruijff E Schmalstieg D Caleydo: Design and Evaluation of a Visual Analysis Framework for Gene Expression Data in its Biological Context Proc of the IEEE Symp on Pac Vis 2010 57 64

[B31] Kim J Cho YJ Do E Choi J Hu G Cadieux B Jung WH A defect in iron uptake enhances the susceptibility of Cryptococcus neoformans to azole antifungal drugs Fungal Genetics and Biology 2012 49 11 955 966 10.1016/j.fgb.2012.08.006 22975303 PMC4706552

[B32] Khodursky AB Peter BJ Cozzarelli NR Botstein D Brown PO Yanofsky C DNA microarray analysis of gene expression in response to physiological and genetic changes that affect tryptophan metabolism in Escherichia coli Proc of the Nat Acad of Sciences 2000 97 22 12170 12175 10.1073/pnas.220414297 PMC17313 11027315

[B33] Gansner ER Hu Y Kobourov S GMap: Visualizing graphs and clusters as maps Proc IEEE Pacific Vis Symp 2010 201 208 10.1109/MCG.2010.101 24807898

[B34] Collins C Penn G Carpendale S Bubble sets: Revealing set relations with isocontours over existing visualizations IEEE Trans on Vis and Comput Graph 2009 15 6 1009 1016 10.1109/TVCG.2009.122 19834166

